# Rethinking professional boundaries: the climate crisis and brain health

**DOI:** 10.1192/bjb.2024.30

**Published:** 2025-02

**Authors:** Ronán M. Conroy, Jeannette Golden, Conor Malone

**Affiliations:** 1 RCSI University of Health Sciences, Dublin, Ireland; 2 St James's Hospital, Dublin, Ireland

**Keywords:** Depressive disorders, anxiety or fear-related disorders, neurodevelopmental disorders, dementias/neurodegenerative diseases, epidemiology

## Abstract

Since climate change affects psychiatric, neurological and neuropsychological disorders, as well as brain development, the Irish Doctors for the Environment working group on mental health has changed its title and remit to brain health. Mental health professionals need to respond coherently and effectively to the climate crisis. This need challenges traditional professional, disciplinary and academic boundaries and demands a holistic, person-centred approach. We propose that meeting this challenge is vital if the public, policy-makers and legislators are to grasp the full extent of the significance of climate's impact on brain health.

Irish Doctors for the Environment (IDE) is a voluntary organisation of Irish doctors and health professionals. We advocate and act to avert the collapse of earth's climate and to reduce the harms to human health that it entails (ide.ie/about/what-we-do). The central message of IDE's campaigning is that the climate crisis is a health crisis, that the same factors that are driving the climate to collapse are having extensive effects right now on human health.

Recently, the IDE working group on mental health took the decision to change its title and remit to brain health. Behind this decision is the realisation that our climate does not distinguish between psychiatric, neurological and neuropsychological disorders, and that environmental destruction has an impact not just on brain disorder, but also on brain development.

The principal agent of climate change is air, or rather the pollutants that human activity has put into the air. In IDE we frequently encounter the belief that climate change is scheduled to happen in 2050 – a long time in the future. We must point out, sadly, that every time a person breathes, they inhale a litre and a half of the climate crisis.

When we think of air pollution and health, there is the immediate association with lung disease, with current estimates linking it to 500 000 lung cancer deaths and 1 600 000 deaths from chronic obstructive pulmonary disease (COPD) worldwide annually.^1^ In addition, its effect on the incidence of asthma in children is of particular concern, with estimates of 35% of cases in Europe, for example, attributable to air pollution.^2^ However, its effects are considerably more far-reaching: there are adverse effects of air pollution on risk and prognosis in cardiovascular disease, cancer, autoimmune disease, irreversible blindness,^3^ diabetes, osteoporosis and bone fracture, reproductive health and childhood cancers among others (for a more comprehensive overview see Table 1 in Schraufnagel et al^1^). The multiple pathways between air pollution and multiple adverse health outcomes involved have required the development of a new paradigm of classification.^4^

## Climate and brain health

When we think of mental health, we tend to think only of the effect of changed climate and weather on psychiatric disorders. Events such as fires, floods and storms can cause deep psychological trauma. Longer-term changes, such as drought and desertification, crop failure and famine, can lead to social displacement and societal violence, which will have an impact on rates of psychiatric disorder.^5^ But the more immediate and graver threat is posed, once again, by the key driver of climate collapse: air pollution.

Studying the link between exposure to air pollution and the risk of acute psychiatric disorder is complex because those most exposed to pollution are the most disadvantaged members of society, who are also least likely to be diagnosed and treated^6^ – yet another reason, we note, for placing poverty and social inequality firmly at the centre of the psychiatric agenda.^7^ Nonetheless, a recent systematic review concludes that ‘extant literature suggests that air pollution is associated with increased depressive and anxiety symptoms and behaviors, and alterations in brain regions implicated in risk of psychopathology’.^8^ Short-term exposure to airborne particulate matter containing particles <2.5 μm in diameter (PM_2.5_) is associated with increased emergency department visits for psychiatric emergencies,^9^ and even short-term exposure to air pollution has adverse effects on cognitive performance that a *Nature Aging* editorial review described as ‘quite startling’.^10^ Long-term exposure, predictably, is associated with increased incidence of schizophrenia spectrum disorder, depression and anxiety disorders.^11^ Added to this, the increase in heatwaves and extreme heat have implications for cognitive performance affecting areas such as education, industry and administration.^12^

The evidence of the impact of air pollution on brain structure and function suggests that risk of neurodegenerative disease may also be increased. And, unsurprisingly, there are robust links between air pollution and cognitive decline and dementia^13,14^ and Parkinson's disease.^15^ Although the mechanism is not clear, it is likely that the same pathway is involved as in cardiovascular disease and stroke: a pro-inflammatory effect.^16–18^

Although the links between air pollution and neurodegenerative disease are of concern, the most disturbing finding is the association with neurodevelopment. Exposures *in utero* to fine particulate matter (PM_2.5_) and nitrogen dioxide (NO_2_) are associated with deficits in cognitive and psychomotor development in children. These effects are evident as early as 15 months,^19,20^ and are evident across a wide range of real-life indices of cognitive performance.^21–25^ Air pollution *in utero* and subsequently is also linked with risk of autism spectrum disorder^26,27^ and attention-deficit hyperactivity disorder.^28^

A disturbing aspect of these associations is that they indicate that widespread and pervasive effects on neurodevelopment are already affecting children around the world. We do not yet know the long-term implications of these effects for mental health and risk of psychiatric and neurodegenerative disease in later life, but frankly, based on the observed effects so far, the outlook appears grim. This concern is echoed by the recent reports of the European Environmental Agency, which has just issued a report detailing the impact of air pollution on children.^29^

Nor is air pollution the only way in which the fossil fuel industry is adversely affecting both climate and health. The noise generated by air, road and rail transport has an impact on both cardiovascular^30^ and mental health.^31^ And, more ominously, it is linked to cognitive impairment in middle-aged adults and to poorer school attainment in children.^32^ Research into transport noise exposure is far from complete, so the current literature may well underestimate the scope and significance of its effects. The same applies to microparticle pollution associated with wear of vehicle tyres (tyres are, we remind the reader, another product of the petrochemical industry). Although it is clear that tyres are an important source of inhaled pollutants,^33^ further research is needed to clarify the independent contribution of these pollutants to the global burden of disease caused by air pollution.

## A challenge to traditional boundaries

It is clear from the associations presented above that the distinction between psychiatric disorder, impaired neurodevelopment and neurodegenerative disorders is unhelpful when tackling the impacts of the climate crisis on health and quality of life. All of these disorders are significantly associated with exposure to the key driver of climate collapse – air pollution. They are occurring in the same brains of the same people, and are strongly correlated. For this reason, IDE has reconfigured the working group on climate and mental health to focus on climate and brain health.

This broadening of scope has several advantages. The first is that it switches the focus from the classification of medical disorders rooted in a mind/body dichotomy to a focus on the health of the person. The brain and its function in the context of the whole person as a member of society is the paradigm that provides a unifying framework. Second, mental disorder is a stigmatising term, which many people would be reluctant to apply to themselves. However, everyone has a brain and most people are rightly concerned about their risk of neurodegenerative disorder in later life. So the focus on brain health allows the climate message to reach a broader audience. Critically, many of us have children whom we cherish more than we do ourselves. The realisation that exposure to air pollution begins damaging the brain even before your child is born is one that no parent can accept – the present authors included!

The change of perspective from mental health to brain health also exposes one of the weaknesses of the health professions in mounting an appropriate response to the climate crisis. The compartmentalisation resulting from medical specialism means that there is no professional body to advocate for brain health. We in IDE believe that there should be no need for such an environmental organisation of health professionals. Our professional bodies should advocate for the health of the population as part of their societal role. However, even if the various bodies were to embrace this role wholeheartedly, there would still be the problem of transdisciplinary cooperation. The distinctions between health specialties that have their roots in the past are not fit for purpose in the current crisis.

The climate crisis is a brain health disaster that threatens all of us and – most worryingly – threatens the future of our children. The situation demands urgent coordinated action by healthcare professionals and organisations.^34^ The Royal College of Psychiatrists has already declared a climate and ecological emergency that is having an impact on the mental health of populations, including the amplification of existing inequalities.^35^ And, writing in this journal, Romeu has proposed that psychiatrists have a moral responsibility to take action.^36^ The bold step of placing this action in the wider context of brain health will promote a coherent and holistic vision of the problem and better able us to take concerted action to tackle it.

All health professions urgently need to mount a widespread response to this crisis that is coherent and transdisciplinary. And such a transdisciplinary framework stands to benefit quality of care and coherence of research and teaching. What are we waiting for?

## Data Availability

Data availability is not applicable to this article as no new data were created or analysed in this study.

## About the authors

**Ronán M. Conroy** is Emeritus Professor in the Department of Public Health and Epidemiology at the Royal College of Surgeons in Ireland (RCSI) University of Medicine and Health Sciences, Dublin, Ireland. **Jeannette Golden** is a consultant old age psychiatrist with the Psychological Medicine Service at St James's Hospital, Dublin, Ireland. **Conor Malone** is a registrar in health informatics at St James's Hospital, Dublin, Ireland.
